# Crohn’s Disease: A Surgeon’s Perspective

**DOI:** 10.4103/1319-3767.74430

**Published:** 2011

**Authors:** Fazl Q. Parray, Mohd Lateef Wani, Akram H. Bijli, Natasha Thakur, Ifat Irshad, Nayeem-ul-Hassan

**Affiliations:** Department of General Surgery and Allied Specialities, Sher-i-Kashmir Institute of Medical Sciences, Soura, Srinagar, India

**Keywords:** Colonoscopy, Crohn’s disease, strictureplasty

## Abstract

Crohn’s disease (CD) is known for wide anatomic distribution, different presentations, life-threatening complications, and multiple modalities of management. Its multiple implications are still unaddressed. Since all the patients do not show a good response to medical modalities of treatment, a significant percentage of these patients are referred to the surgeon for the palliation of complications or for the ultimate curative treatment. Since most surgeons come across such patients only rarely, it is sometimes difficult for them to choose the appropriate procedure at the time of need. Moreover, the various surgical modalities available for the different presentations and complications of the disease have not been adequately discussed. The aim of this review is to offer insight and a detailed account of the management of CD from a surgical perspective. This review offers an overview of the various surgical options available, their utility in context, and an approach to various scenarios of complicated CD.

Crohn’s disease (CD) is characterized by recurring episodes of suppurative inflammation in any part of the gastrointestinal tract from the mouth to the anus. The inflammation is transmural and can result in strictures, microperforations, and fistulae. The inflammation is non-contiguous and can thus produce skip lesions throughout the bowel. CD is a lifelong disease in the etiology of which genetic, immunological, and environmental factors are involved. It is seen predominantly in the developed countries of the world. The precise etiology is not known and therefore a causal treatment is not yet available. The incidence of CD in the US is 4/100000. As the disease is chronic, the prevalence is much higher: around 80–150/100000.[1,2] CD can affect any age-group but typically affects young adults, with the Peak age for manifesting the disease i.e peak incidence in 15-25 year age group; it is extremely rare under the age of 6 years. There is a small female prepondence; however, a contradictory preponderance has been reported in some populations.[3]

## ETIOPATHOGENESIS

The exact etiology of CD is not known. Many possible causes have been the subject of speculation and investigation.[4] Scientific research into the molecular biology of CD has begun to give some insights into the genetics of this condition, but has not yielded total clarity. However, a reasonable hypothesis for pathogenesis of CD suggests that an as yet unidentified antigen (possibly a mycobacterium, paramyxovirus, or components of cigarette smoke) activates resting macrophages to release a wide variety of cytokines in susceptible individuals.[5] Cytokine is a collective term for a group of low-molecular-weight peptides that are active at very low concentrations and bind to specific receptors to produce autocrine, paracrine, and endocrine effects. The most abundant cytokine is interleukin-1 (IL-1), which not only produces diarrhoea but also acts as a pyrogen. These cytokines serve to stimulate the immune system and cause an inflammatory reaction, thus producing tissue damage in intestinal mucosa.[5] To summarize, CD is characterized by an exaggerated and destructive mucosal immune response. The tissue injury is likely to be initiated by diverse genetic and immunological factors that are modified by environmental influences, including microbes and their products.

## PATHOLOGY

The earliest gross manifestation of CD is the development of small mucosal ulcerations called aphthous ulcers. These appear as red spots or focal mucosal depressions, typically occurring over submucosal lymphoid aggregates.[6] They enlarge, become stellate, and coalesce to form longitudinal mucosal ulcerations. These linear ulcerations always occur along the mesenteric aspect of the bowel lumen. Progression of these linear ulcerations leads to a serpiginous network surrounding islands of edematous mucosa, producing the classic ‘cobblestone’ appearance. Mucosal ulcerations may penetrate through the submucosa to form intramural channels that can bore deeply into the bowel wall and create sinuses, abscesses, or fistulas.

The inflammatory process progresses to extend through all the layers of the bowel wall. The inflammation in CD also involves the mesentery and regional lymph nodes, such that the mesentery may become massively thickened. With early acute intestinal inflammation, the bowel wall is hyperemic and boggy. As the inflammation becomes chronic, fibrotic scarring develops and the bowel wall becomes thickened and leathery in texture.

Histopathologic examination of specimen from CD typically demonstrates transmural inflammation characterized by multiple lymphoid aggregates in a thickened submucosa. Lymphoid aggregates may extend beyond the mucosa and be found within the muscularis propria.[6] The presence of well-formed lymphoid aggregates in an edematous fibrotic submucosa is a classic histological feature of the disease. Another charecteristic microscopic feature of CD is the presence of non-caseating granulomas. Non-caseating granulomas are a valuable diagnostic feature of CD but are seen in only 50% of resected specimens and are rarely seen on endoscopic biopsies. The presence of granulomas does not correlate with disease activity, as areas of active inflammation are no more likely to contain granulomas than areas of quiescent disease.[7]

## PATTERN OF DISEASE

CD can be categorized into three general types according to the predominant gross manifestation of the disease: stricturing disease, perforating disease, and inflammatory disease.[8,9]

### Stricturing disease

In this type, the inflammation results in formation of fibrotic scar that constricts the lumen of intestine, resulting in fibrostenotic lesions. These fibrostenotic lesions do not respond to medical therapy. These patients usually present with features of intestinal obstruction and often require surgical intervention.

### Perforating disease

Sinus tracts, fistulae, and abscesses are characteristic of perforating disease. Sinus tracts developing from mucosal ulcerations penetrate the muscularis propria and give rise to abscesses or fistulae. The sinus often bores through areas of adhesion and therefore abscess formation or fistulization to other structures occur much more often than does free perforation into the peritoneal cavity.

### Inflammatory disease

The inflammatory pattern of CD is characterized by mucosal ulceration and bowel wall thickening. In the small intestine, this pattern often gives rise to obstructive symptoms, whereas when the colon is involved there is diarrhea. Of the three patterns of disease, the inflammatory pattern is much more likely to respond to medical therapy.

## CLINICAL FEATURES

The clinical presentation and symptoms of CD vary greatly depending on the segment of intestine involved.[10] CD activity index (CDAI) is used in all clinical trials to measure disease activity[11] [Table 1]. A score of less than 150 indicates a clinical remission and a score of over 450 indicates severely active disease.[11]

**Table 1:** Crohn’s disease activity index

Variable	Scale	Weight
Liquid or very soft stool	Daily stool count is summed for 7 days	2
Abdominal pain	Sum of 7 days of daily ratings as 0=none, 1=mild, 2=moderate, 3=severe	5
General wellbeing	Sum of 7 days of daily ratings as 0=generally well, 1=slightly below par, 2=poor, 3=very poor, 4=terrible	7
Features of extraintestinal disease	Presence of any of the following in the previous 7 days:	20 each
	Arthritis or arthralgia	
	Skin or mouth lesions (erythema nodosum, aphthous ulcers, pyoderma gangrenosum) Iritis or uveitis Anal fi ssures, fi stulas, perianal abcess Other external fi stulas Fever >100°F	
Opiates for diarrhea	0=No, 1=Yes	30
Abdominal mass	0=None, 2= Questionable, 5=definite	10
Hematocrit	Men 47% hematocrit	6
	Women 42% hematocrit	
Body weight	100 × [1-(body weight/standard weight)]	1

### Crohn’s disease of upper gastrointestinal tract

CD of the upper gastrointestinal tract usually presents with nausea, vomiting, dysphagia, or odynophagia.[12] Oral CD presents with aphthous ulcers over the hard palate. Esophageal CD is very rare and is believed to be more common in children than adults.[13] Isolated involvement of the esophagus is almost never seen; there is always associated CD elsewhere in the gastrointestinal tract. Esophageal involvement presents with dysphagia or odynophagia. Gastroduodenal CD presents with postprandial fullness, nausea, vomiting, and features of delayed gastric emptying.

### Crohn’s disease of the small intestine

The predominant symptom of small bowel CD is abdominal pain, which occurs in 90% of patients.[10] Other common symptoms are weight loss and anorexia. Weight loss may be the result of avoidance of food or of malabsorption. Some patients may develop an abdominal mass as a result of abscess or phlegmon formation. Evidence of fistulization to the skin, bladder, or vagina may be noted in some patients.

### Crohn’s disease of the large intestine

Crohn’s involvement of the colon typically presents with diarrhea that may or may not be bloody. Abdominal pain and fever, often exacerbated by bowel movements, is a usual presentation of Crohn’s colitis. Stricturing disease of the colon can give rise to colonic obstruction. Fistulizing disease can give rise to abscess formation and fistulas. Toxic megacolon can occur with CD, but this severe complication is rarely seen, unlike as is seen in ulcerative colitis.[14]

### Perianal Crohn’s disease

Approximately 40% of Crohn’s patients will develop perianal manifestation of the disease. Perianal CD can present with perianal fistulae, abscesses, hypertrophied skin tag, fissures, or perianal scarring.[15,16]

### Extraintestinal Crohn’s disease

Extraintestinal manifestations, when present, produce symptoms that can be more severe than those of the primary intestinal disease and typically correlate with the activity of intestinal CD. A variety of extraintestinal manifestations can occur in CD. These include ocular, dermatological, hepatobiliary, and joint disorders.[17,18] Ocular manifestations of CD include uveitis and episcleritis.[19] Cutaneous manifestations of CD include erythema nodosum and pyoderma gangrenosum. Joint disorders such as ankylosing spondylitis, sacroiliitis, and seronegative polyarteritis can occur. Patients with CD are also at risk of developing primary sclerosing cholangitis.

## INVESTIGATIONS

### Small bowel radiography

Small bowel contrast study, either follow-through or enteroclysis, is the best means for assessing small bowel CD.[20–23] The radiographic abnormalities are often distinctive.[24] The radiographic features include ulcers, cobblestoning, strictures, string sign, fissures, loss of haustral markings, etc. However, it is still possible to miss certain fistulae and strictures.[25–27]

### Colonoscopy

This procedure allows direct inspection of the mucosa, and biopsy can be taken. Push colonoscopy can be done to evaluate the ileum. Characteristic findings on colonoscopy include longitudinal ulcerations, aphthous ulcers, skip lesions, pseudopolyps, and strictures.[24]

### Capsule endoscopy

This is a new tool for the diagnosis and evaluation of CD.[28,29] A small camera constructed within a capsule-size casing is swallowed and images from the camera are transmitted to a small electronic receiver worn by the patient. The image from the capsule endoscopy can detect subtle mucosal lesions that may not be apparent on small bowel x-rays. This diagnostic modality should be reserved for those cases in which there is a substantial diagnostic uncertainty. Small bowel strictures should be excluded prior to the capsule endoscopy, as the capsule may fail to pass through areas of narrowing and result in intestinal obstruction.

### Computed tomography

Computed tomography (CT) is not routinely necessary for the diagnosis of CD. CT, however, is very useful in identifying the complications associated with CD.[30,31] CT can readily identify thickened and dilated intestinal loops, inflammatory masses, or abscesses. CT is the most sensitive modality for identifying the presence of an enterovesical fistula, which is identified by the presence of air within the urinary bladder.

### CT enterography, MR enterography

These are new modalities for investigating CD. These can be used three dimensionally to locate the area of intensity of the disease.

## DIFFERENTIAL DIAGNOSIS

The principle alternatives in the differential diagnoses of CD are appendicitis, bowel tuberculosis, small bowel cancer, non-steroidal enteropathy, diverticulitis, celiac sprue, lymphoma, postsurgical adhesions, Behcet disease, ischemic colitis, radiation enteropathy, colitis ulcerosa, and infection enteritis.

## MEDICAL MANAGEMENT

### Goals of treatment

The goals of treatment are:

- Induction of remission
- Maintenance of remission
- Healing of mucosal lining
- Prevention of complications
- Improvement of quality of life
- Reduction or elimination of use of steroids
- Avoidance of hospitalization and surgery
- Restoration and maintenance of nutrition

### Disease definitions

Remission: When CDAI score is <150.

Response: When decrease in CDAI is by 100 points.

Relapse: Flare-up in a patient with established CD in clinical remission or CDAI >150 with increase of >70 points.

Early relapse: Flare-up of disease within 3 months after achieving remission

Infrequent relapse: One relapse per year

Frequent relapse: Two relapses per year

Continuous disease: Persistence of symptoms of active CD, without a period of remission.

Corticosteroid-refractory CD: Active disease despite prednisolone at a dose of up to 0.75 mg/kg/day × 4weeks.

Corticosteroid-dependent: When corticosteroid cannot be reduced below prednisolone 10 mg/budesonide 3 mg within 3 months of starting steroid *or* relapse within 3 months of stopping steroids

Recurrence:

- Reappearance of lesions after surgical resection
- Morphological recurrence - Appearance of new CD lesions after complete resection of macroscopic disease.
- Clinical recurrence - Appearance of CD symptoms after complete resection of macroscopic disease.

Localized disease: Less than 30 cm involved

Extensive disease: More than 100 cm involved (sum of discontinuous areas can also be taken)

### Modalities of medical therapy

#### Corticosteroids

These drugs are only used to induce clinical remission. The majority of patients with small bowel CD will go into clinical remission with prednisolone 0.25–0.5 mg/kg/day. In a patient unable to take oral steroids, methylprednisolone can be given as a daily infusion in the dosage of 40–60 mg.[32,33]

#### Aminosalicylates

Aminosalicylates come in variety of preparations, each designed to deliver the drug to a particular intestinal segment.[34] These include sulfasalazine and 5-aminosalicylic acid (5-ASA) derivatives. 5-ASA compounds inhibit leukotriene production by inhibition of 5-lipooxygenase activity. 5-ASA also inhibits the production of interleukin-1 and tumor necrosis factor (TNF). Aminosalicylates are effective in the treatment of mild to moderate CD. 5-ASA given in a controlled-release preparation is also effective as maintenance therapy to prevent recurrence, after a flare-up of the disease has been effectively managed either medically or surgically.[35–39]

#### Immunomodulators (azathioprine, 6-mercaptopurine, methotrexate, cyclosporin)

These are immunosuppressive drugs that inhibit cytotoxic T cells and natural killer cell function. They are useful in mild to moderate disease.[32,40] Azathioprine in the dosage of 2–2.5 mg/kg/day and 6-mercaptopurine in the dosage of 1–1.5 mg/kg/day will result in 50%–60% response rate in patients with active Crohn’s.[33,41] They can be combined with steroids or biological agents to induce fast remission.

#### Biological therapies (infliximab, adalimumab, and certolizumab pegol)

Infliximab is a chimeric mouse-human monoclonal antibody to TNF. TNF is a proinflammatory cytokine that is believed to be important in the pathophysiology of CD. Infliximab binds to both free and membrane-bound TNF and prevents TNF from binding to cell surface receptors.[35] Clinical trials have demonstrated an 80% response rate with a single dose of infliximab.[42,43] It is important to note that the doses and dosing intervals of infliximab must be individualized, but a typical regimen would include 5 mg/kg of infliximab given with IV at weeks 0, 2, and 6, and then every 8 weeks thereafter. Adalimumab and certolizumab pegol are other congeners.

## SURGICAL MANAGEMENT OF CROHN’S DISEASE

### Goals of surgical treatment

- To provide long lasting symptomatic relief while avoiding excessive morbidity.
- Surgery is only palliative and not curative.
- Complete extirpation of disease should not be the goal.
- Excessive loss of intestinal length should be avoided as far as possible.

### Indications of surgery

1. Failure of medical treatment is the most common indication for surgical intervention.
2. Intestinal obstruction is a common indication for surgery.
3. Fistulas
4. Abscesses and inflammatory masses.
5. Perforation.
6. Hemorrhage.
7. Toxic megacolon.
8. Cancer or suspicion of cancer.
9. Growth retardation.
10. Extraintestinal manifestations.

### Surgical options

#### Intestinal resections

Intestinal resection is the most common surgical procedure performed for CD. Cumulative data indicates that resection of only grossly apparent disease is enough – a wider resection margin does not improve outcome.[44,45] Most cases of CD need only limited resection, which is well tolerated and does not lead to the short bowel syndrome.

#### Anastomosis

There is no consensus over the technique of intestinal anastomosis in CD.[46–50] The argument that side-to-side anastomosis may be better than end-to-end or end-to-side anastomosis is theoretical.[49] Till date no clinical data indicate a benefit of one particular type of intestinal anastomosis over another.[48] In the presence of sepsis, severe scarring, or malnutrition, a proximal loop stoma to protect the anastomosis is wise. However, when performed under elective conditions, anastomosis is safe, with a dehiscence rate of as little as 1%.[51]

#### Stoma formation

A permanent stoma is required for Crohn’s proctitis and severe unrelenting perianal disease. Temporary stoma is required more frequently for protecting the distal anastomosis. Preoperative marking of the stoma site should be done to obtain a satisfactory result and to place the stoma away from the deep skin folds and bony prominences.[52,53] About 25% of the patients with permanent stoma will require revision of their stoma because of one or the other complication of stoma, e.g., hernia, prolapse, or stricture.[54]

#### Strictureplasty

These procedures are best performed when resection is expected to lead to short bowel syndrome, i.e., when there is a long stricture segment, when multiple previous resections have already been done, and when there is a short segment recurrent disease at a previous anastomotic site. There is increased evidence that intensity of the disease decreases at the strictureplasty site and the disease becomes quiescent.[55] It is contraindicated to do a strictureplasty in case of suspicion of malignancy and in stricturing disease of colon.

The most commonly performed strictureplasty is the Heinecke-Mikulicz type,[56,57] which is named after the pyloroplasty technique from which the procedure is derived. This type of strictureplasty is performed in short-segment stricture of 2–3 cm in length.

Finney strictureplasty is done in long-segment stricture of up to 15 cm in length.[56] A very long Finney strictureplasty may, in theory, result in functional bypass, with a large diverticulum that could be at risk for bacterial overgrowth and blind loop syndrome. Jaboulay technique is another method of doing strictureplasty.

Side-to-side isoperistaltic strictureplasty is done in patients with long-segment stricturing disease or multiple strictures grouped close together.[58] The isoperistaltic side-to-side strictureplasty is recognized as an effective means of treating extensive small bowel CD and provides the best option for those cases that would otherwise require extensive intestinal resection with loss of significant length of small bowel.[55,59–61]

Complications of strictureplasty

- Long-term recurrence.
- Risk for malignancy (as diseased segment is retained)
- Hemorrhage from strictureplasty site.

#### Laparoscopy

Indications for laparoscopy in CD are the same as that for open surgery. Laparoscopy for CD has been shown to be safe and feasible; it reduces hospitalization and overall morbidity.[62–75] It is best suited for patients who are:

- Young and otherwise healthy.
- Those who express a wish for operation with minimal scarring.
- Candidates likely to require multiple operations over the lifetime.

However laparoscopy is difficult in:

- Intense inflammation and thickened mesentery.
- Inflammatory masses or abscesses.
- Enteric fistulas.
- Multiple areas of involvement.

Laparoscopy is contraindicated in:

- Critically ill patients and those unable to tolerate pneumoperitoneum.
- Intra-abdominal sepsis - abscess, free perforation, or fistula.
- Difficulty in identifying anatomy.

## MANAGEMENT OF COMPLICATED CROHN’S DISEASE

### Crohn’s disease of duodenum

CD of duodenum usually manifests as a stricturing disease which can be managed by strictureplasty or a bypass procedure, almost never requiring resection.[76–78] When the duodenum is involved in Crohn’s fistula, it is usually the result of disease of the distal segment; typically, the distal ileum fistulizes into an otherwise normal duodenum.[79] Stricturing disease of first three portions of the duodenum is managed by Heineke-Mikulicz type strictureplasty, whereas stricture of the fourth portion can be managed by Finney strictureplasty.[80] A long, rigid, and unyielding duodenal stricture can be managed by a bypass procedure, viz. side-to-side retrocolic gastrojejunostomy.[81] However, highly selective vagotomy should be performed in these patients to lessen the likelihood of stomal ulceration.[81] Strictures limited to the third and fourth portion of the duodenum can be managed by Roux-en-Y duodenojejunostomy to the proximal duodenal segment, with the advantage of bypassing the stricture, and eliminating the risk of ulceration and the need for vagotomy.[79]

Most of the duodenal fistulae are small and asymptomatic; larger fistulae may result in diarrhea and malasorption. Most of the duodenal fistulae can be managed by resection of the primary CD, with primary closure of the duodenal defect. However, large fistulae should be managed by resection and closure with Roux-en-Y duodenojejunostomy or with a jejunal serosal patch.[82,83]

### Crohn’s disease of small bowel

- Complete small bowel obstruction: An acute episode of small bowel obstruction (a first episode) is managed by nasogastric decompression, intravenous hydration, and steroid therapy; surgery is rarely required.[81] However, elective surgery should be done once the acute episode is over, with the advantage that surgery is performed in safer conditions, with less bowel distension and edema. When the acute episode does not respond to a conservative line of treatment, surgery should be performed with strong suspicion of malignancy as benign small bowel obstruction in CD usually responds to conservative management.
- Iliosigmoid fistula: About half of the ileosigmoid fistulas from CD are not recognized prior to surgery.[84] Seventy-five percent of iliosigmoid fistulas can be managed by simple division of the fistulous tract and resection of ileal disease.[26,84] The remainder require resection of the sigmoid colon. Sigmoid colon resection is necessary when primary closure of the fistula is at risk due to poor healing. This is the case when the sigmoid is also involved in CD, when the fistulous opening is particularly large, or when there is extensive fibrosis extending along the sigmoid colon. Also, fistulous tracts that enter the sigmoid colon in proximity to the mesentery can be difficult to close and often require resection and primary anastomosis.
- Iliovesical fistula: This is seen in about 5% of the patients of CD. Hematuria and feculuria are diagnostic signs of iliovesical fistula, though these signs are seen in only about one-third of patients.[85] Air within the bladder on computed tomography is the best evidence of an enterovesical fistula. As many as 60% of the patients of enterovesical fistulae have iliosigmoid fistula.[26] Hence, enterovesical fistula is a marker of complex fistulizing disease. Surgery is generally needed in these patients, especially when there is pyelonephritis with potential for deterioration of renal function. Surgical treatment of ileovesical fistulae requires resection of the ileal disease, with closure of the bladder defect. Decompression of the bladder with an indwelling Foley catheter should be continued postoperatively until the bladder is confidently healed without leaks. A cystogram taken on postoperative day 5 is a convenient means for confirming the seal of the bladder repair and the safety of removing the Foley catheter.
- Abscess: In about one-third of cases, clinical signs of abscess are absent and they are discovered only on laparotomy. When there is a mass on clinical examination, CT should be done, as 50% of tender masses will harbor an abscess within.[86] In case of a small abscess, resection of the diseased segment along with mesentery should be done. In case of a large abscess, CT-guided aspiration should be done, which is very effective in controlling sepsis and for healing of the abscess.[87,88] Percutaneous drainage often leads to enterocutaneous fistula, which can be initially managed conservatively. If conservative management fails, surgical resection of the affected segment may be necessary.[89]
- Perforation: Most perforations in CD occur in the terminal ileum proximal to the stenotic lesion.[81,90] Diagnosis is usually made by identifying the presence of free gas on CT or abdominal x-ray. Perforation is an absolute indication for laparotomy. Resection of the diseased segment with exteriorization of the proximal bowel as an end ileostomy should be done. In case of primary repair/anastomosis, a proximal diversion ileostomy should be done as there is a high risk of anastomotic breakdown.
- Hemorrhage: Massive hemorrhage due to CD is very rare. Indolent, episodic, chronic bleeding can be managed by intermittent transfusion. Massive hemorrhage occurs due to erosion of a single vessel. Small bowel angiography may localize the bleed.[91] Recurrent hemorrhage after spontaneous stoppage of the first episode is common. Elective resection of the diseased segment should be carried out after the first episode of hemorrhage.
- Enterocutaneous fistula and vaginal fistula: These complications need surgical treatment and are often a manifestation of an underlying complex disease.

### Crohn’s disease of colon

- Cecal disease: This almost always occurs with terminal ileal disease, with the ileal component of the disease being predominant. Treatment includes resection of gross disease with ileo-ascending anastomosis. In patients with previous intestinal resections, another option for surgical treatment is side-to-side ileocolic enteroplasty (Poggioli technique).
- Right-sided colitis: This can occur alone but is more commonly seen along with disease of the terminal ileum. A standard right hemicolectomy with ileotransverse anastomosis is the surgical treatment. However, an omental patch should be placed between the anastomosis and the duodenum to prevent fistula formation.
- Extensive colitis with rectal sparing: This occurs in about 20% of individuals suffering from Crohn’s colitis. If the rectum is truly free of disease, then a total abdominal colectomy with ileorectal anastomosis can be performed when fecal continence is adequate and the patient does not have extensive perianal septic complications. Up to 50% of patients who undergo an ileorectal anastomosis for colonic CD will ultimately require a proctectomy with permanent ileostomy due to poor bowel function with incontinence, or due to recurrence of disease in the rectum.[92]
- Proctocolitis: Extensive involvement of the colon and rectum requires total proctocolectomy with permenant ileostomy, almost in all cases in a single sitting. However, patients with perianal septic disease require two sittings for the same. Restorative procedures like ileal pouch–anal anastomosis or continent ileostomy should not be offered to patients with Crohn’s colitis because of the recurrent nature of the disease, high risk of fistula, and increased bowel frequency. However, there is a specific pattern of CD that is at low risk for recurrence after ileoanal anastomosis.[93,94]
- Proctitis: CD limited to the rectum is rare. Surgical treatment of Crohn’s proctitis mandates proctectomy with a permanent stoma. The need for resection of the normal proximal colon is controversial.
  - Total proctocolectomy with end ileostomy should be done for disease limited to the rectum and distal colon and in the young with no history of small bowel disease, as colorectal disease without small bowel involvement is unlikely to recur after proctocolectomy.[14]
  - Abdominoperianal resection with end-sigmoid colostomy should be done in older patients, patients who have undergone prior resection of small bowel disease, and elderly patients.
- Segmental colitis: The optimal management of segmental colitis depends not only on the location of the disease but also on its extension and status of perianal complications. Segmental involvement of the right colon should be managed by simple right hemicolectomy with ileo-transverse anastomosis. Involvement of the transverse colon should be managed by extended right hemicolectomy, which is to be preferred over segmental transverse colectomy as it has low risk of recurrence and avoids colo-colic anastomosis. In the descending or sigmoid colon, appropriate surgery is more controversial. Segmental resection with colo-colic anastomosis is performed with good results.[95,96] The advantage of preserving the absorptive capacity outweighs the risk for recurrence.
- Perianal CD: Manifestations include abscesses, fistulas, fissures, anal stenosis, and hypertrophic skin tags.[97,98] It is seen in one-third of the patients of intestinal CD.[42] However, whether activity of perianal Crohn’s parallels the activity of intestinal Crohn’s is still controversial.
  - For abscess, incision and drainage is often required.
  - For low-lying uncomplicated submucosal or intersphincteric fistulas, an initial trial of antibiotics (metronidazole or ciprofloxacin) is indicated; if it fails, surgical fistulotomy is performed.[99,100] Fistulotomy should be performed only in superficial fistulae and the use of non-cutting seton is always preferable.
  - For complex fistulas (supra- and trans-sphincteric) fistulotomies and cutting setons should not be used; instead, medical management (6-mercaptopurine, azathioprime, infliximab) should be used. If it fails, proper placement of non-cutting setons is most appropriate.[101–103]
  - Sphincter-preserving surgery, such as flap closures, fibrin glue, or plugs, can also be an option in the management of perianal CD.

### Postoperative maintenance therapy

The risk of recurrent disease can be lessened by postoperative maintenance therapy. The most common agents used for postoperative maintenance therapy are controlled-release 5-aminosalisylic acid and 6-mercaptopurine.[36] The use of 5-aminosalicylic acid is associated with low risk of adverse effects but requires taking up to 16 tablets a day. In comparison, 6-mercaptopurine is less expensive, requires just one daily dose, and is more effective.[36] However, it causes bone marrow suppression and there is a need for monitoring blood cell counts. The decision for postoperative maintenance therapy should be individualized for each patient.
